# A novel calibration technique to obtain high resolution 3D T1 Maps for Infarct characterization

**DOI:** 10.1186/1532-429X-18-S1-P101

**Published:** 2016-01-27

**Authors:** Paras Parikh, Jason Ng, Michael Markl, Timothy Carroll, Jeffrey J Goldberger, Brandon C Benefield, Justin Ng, Daniel C Lee

**Affiliations:** 1 grid.465264.7Biomedical Engineering, Northwestern University, Chicago, OH USA; 2 grid.465264.7Feinburg School of Medicine, Northwestern University, Chicago, IL USA

## Background

Myocardial T1 mapping enables quantification of extracellular volume fraction (ECV), which correlates with myocardial fibrosis and is predictive of adverse cardiac events. Quantitative T1 mapping is highly useful in detecting scar tissue in patients with prior myocardial infarctions or those with other cardiomyopathies. The Modified Look-Locker inversion recovery (MOLLI) sequence is a clinical standard for T1 mapping, however it is limited by low spatial resolution which prevents it from being used for infarct characterization. In this study, a novel technique to convert high resolution 3D phase sensitive inversion recovery (3D-PSIR) images into high resolution T1 maps by calibration with low-resolution MOLLI T1 maps.

## Methods

From a phantom study using 11 agarose vials doped with different concentrations of Ni^+2^, an exponential relationship was derived between signal intensity and T1 using a 3D-PSIR segmented Turbo FLASH and MOLLI acquisition, respectively. 9 canines with chronic infarcts (ranging from 12-14 weeks) were scanned on a 1.5 T Siemens Avanto scanner. A 3D-PSIR (covering the entire left ventricle) and a single long axis MOLLI were collected prior to contrast injection and 15-20 minutes after contrast injection (Gd-DTPA). The MOLLI and 3D-PSIR images had resolutions of approximately isotropic in-plane 1.3-1.4 mm × 8.0 mm and 1.4 × 1.4 × 1.4 mm, respectively. Six regions of interest (3 in the myocardium, 2 in the blood pool, and 1 in the liver) were drawn in the MOLLI image and the corresponding 3D-PSIR image to determine the exponential relationship between signal intensity and T1 based upon the Bloch equation. The high resolution reconstructed T1 maps of the left ventricle from the 3D PSIR images were then compared to the MOLLI T1 maps based on 10 ROI's drawn from the anteroseptum to the inferolateral wall of the left ventricle in the long axis slice. Mean T1 values were collected for each ROI and a linear regression was used to test the association of T1 values from the MOLLI image to the T1 values from the reconstructed T1 maps.

## Results

The correlation between T1 values of MOLLI and reconstructed T1 maps showed an R^2^ value of 0.938 for all nine dogs (see figure, p<.009). The figure also illustrates a clearer delineation of the myocardial and scar boundaries in the calibrated 3D-PSIR based T1 maps when compared to the MOLLI T1 maps.

## Conclusions

High resolution 3D maps provide a resolution of 2.74 mm^3^ for the T1 reconstructed maps versus 15.7 mm^3^ for the 2D MOLLI T1 maps. The T1 maps developed using this calibration technique can provide a better understanding of the 3D architecture of scar in patients with prior myocardial infarctions and can further delineate normal tissue versus scar tissue within infarct zones.

**Figure 1:**
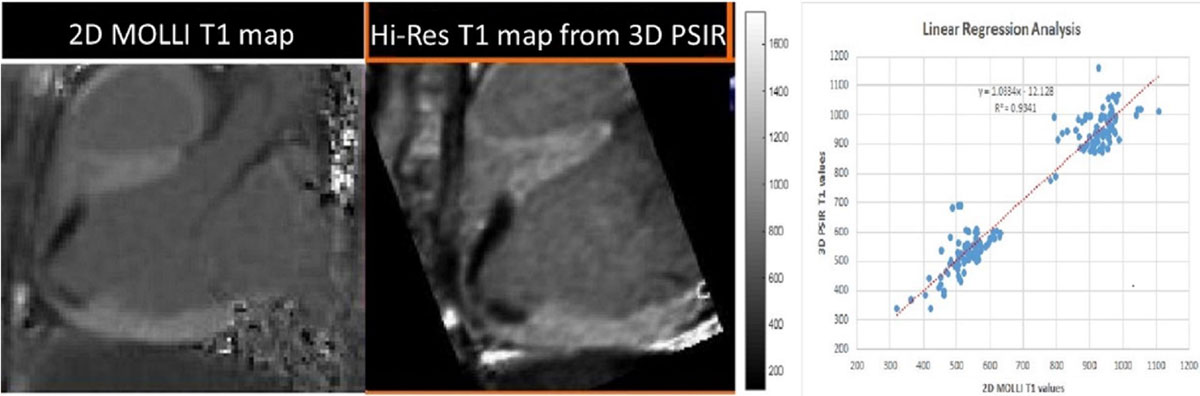
Figure 1

